# Clinicopathologic analysis of primary gastroenteropancreatic poorly differentiated neuroendocrine carcinoma; A ten year retrospective study of 68 cases at Moffit Cancer Center

**DOI:** 10.12669/pjms.36.2.1336

**Published:** 2020

**Authors:** Mulazim Hussain Bukhari, Domenico Coppola, Aejaz Nasir

**Affiliations:** 1 Prof. Mulazim Hussain Bukhari, MBBS, DCP, MPhil, FCPS, CHPE, PhD. Department of Pathology, UCMD, University of Lahore, Lahore, Pakistan; 2 Domenico Coppola, MD. Emeritus Professor of Inter-disciplinary Oncology, Department of Anatomic Pathology, H. Lee Moffitt Cancer Center Tampa, FL, USA; 3 Aejaz Nasir, MD, MPhil, FCAP. Chief Pathologist, BJ’s Diagnostic & Precision Oncology, Tampa, FL, USA

**Keywords:** Etoposide, Gastrointestinal cancer, Neuroendocrine cancer, Pathology Platinum

## Abstract

**Objective:**

To assess clinicopathological characteristics of primary gastro-entero-pancreatic poorly differentiated neuroendocrine carcinomas (GEP-PDNECAs) and evaluate overall survival in patients treated with systemic platinum and etoposide therapy.

**Methods:**

A detailed retrospective review of clinico-pathologic data (1999-2009) on 68 consecutive adult patients with primary GEP-PDNECAs was carried out, from H Lee Moffit Cancer Center and Research Institute, Tampa, Florida; USA, based on electronic patient records, specialty consultation files, tumor registry, social security index and pathology archives. All available tumor slides were reviewed and subtyped by neuro-endocrine pathologists. Clinicopathologic data and patient survival were analyzed.

**Results:**

Of 68 patients 41 were males and 27 females with a mean age of 42 years (range: 25-76 years). Regarding the site of origin, 39 patients were of the colorectal location, 19 from the pancreas, 04 from small intestines, 03 from stomach and 03 were multi-focal from colon, small intestine and pancreas. Sixty three of 68 (93%) patients presented with lymph node/distant metastases. Of 68 tumors 37 (54%) were classified as small cell carcinoma (SCCA), 16 (24%) large cell carcinoma (LCCA), 5 (7%) mixed small and large cell (MSLCCA) and 10 (15%) poorly differentiated carcinoma with neuroendocrine features (PDCA-NEF). Neuroendocrine differentiation was confirmed by positivity for chromogranin in 38/65 (55%), synaptophysin in 62/67 (92%) and CD56 in 17/21 (81%) cases. One neuroendocrine marker was positive in 22/68 (32%), 2 in 40/68 (59%) and all 3 were positive in 9/68 (13%) cases. Fifty-eight of 68 (85%) patients were treated with platinum and etoposide. Overall patient survival at 1, 3 5 and 10 years was 85%, 40%, 16% and 1.5% respectively. Patient survival was independent of age (r= 0.1022), sex (r= -0.909) and histologic tumor subtype (r=0.1028) (p= 0.128) but was related to distant metastases (r=0.306; p=0.0383).

**Conclusions:**

GEP-PDNECA occurred in many part of the GI tract, most commonly in the colorectal region. Positivity of neuroendocrine markers was variable, which helped to confirm neuro-endocrine differentiation and to avoid under-diagnosis of GEP-PDNECA, especially in metastatic setting. Overall prognosis of GEP-PDNECA patients following platinum and etoposide therapy in our series was relatively favorable but remained poor in the presence of distant metastases.

## INTRODUCTION

Gastroenteropancreatic neuroendocrine tumors (GEP-NETs) are relatively rare and complex neoplasms that present many clinical challenges. They arise from the diffuse neuroendocrine system, and gastrointestinal tract is one of the most common locations of the tumors. These are heterogeneous group of neoplasms characterized by differences in embryologic, biologic, and histopathologic aspects. GEP-NETs have traditionally been divided into foregut (esophagus, stomach, proximal duodenum, liver and pancreas), midgut (distal duodenum ileum, jejunum, ascending colon and proximal two thirds of transverse colon) and hindgut tumors (distal third of transverse colon, descending colon, sigmoid colon and rectum).^1^-^4^

Although NETs are rare tumors but their frequency is continuously increasing, possibly due to the greater awareness, early detection and new modalities for their diagnosis and treatment. Gastroenteropancreatic NETs constitute about 2% to 3% of all gastrointestinal malignancies. The prognosis for the distant-stage gastrointestinal NETs and pancreatic NETs in particular has improved over time, especially reflecting improvement in these modalities. Prognosis of the patients with GEP-NETs depends on stage and histology. Most NETs have indolent behavior despite presence of distant metastasis with a poor 5-year survival. Patients with well- and moderately-differentiated distant metastases have a 5-year survival probability of 35%; conversely, in patients with poorly differentiated distant metastases, the 5-year survival probability drops to only 4%.^5^

These tumors are non-specific in their presentation and slow in onset of symptoms contributing to delayed diagnosis. Unfortunately, when these patients are finally diagnosed with an NET, many will already have metastases to lung, liver, lymph nodes and bones. The most common site of metastases being the regional lymph nodes.^6^ MRI and CT scan can help in localization and clinical staging of patients with NET primaries and metastases.^7^-^9^

Histologically, these are divided into three grades on the basis of clinical behavior, histology, and proliferation rate: Well differentiated, G1, low grade when the Ki67 index is less than 2%, Intermediate group G2 with Ki67 2-20% and mitosis also 2-20%/10HPF, and poorly differentiated or G3, high grade neuroendocrine carcinoma with mitosis >20/10HPF and Ki67 more than 20%. Their clinical behavior depends upon the site of origin. The NETs arising from the small intestine have more malignant potential but will show slow progression at their metastatic site. On the other hand, NETs of stomach and colon often have a low tendency to metastasize but can progress rapidly once they become metastatic.^5^,^10^

The diagnosis of these tumors needs a multidisciplinary coordination, among oncologists, surgeons, radiologists and pathologists. Results from histopathology, hormonal analysis, and imaging are required to establish a comprehensive diagnostic approach. Patient management depends upon tumor site, size, grade, stage, patient’s symptoms, age and comorbidities. Surgery is better option with focusing on margin-negative resection and adequate lymphadenectomy is the only curative treatment modality to date. In disease with metastasis, surgery plus chemotherapy may improve prognosis. The treatment options are rapidly expanding and many patients with neuroendocrine tumours may have increased survival time with improved symptom control and quality of life.^11^

Our objective was to assess clinicopathological characteristics of primary gastro-entero-pancreatic poorly differentiated neuroendocrine carcinomas (GEP-PDNECAs) and evaluate overall survival in patients treated with systemic platinum and etoposide therapy.

## METHODS

With the approval of Institutional Review Board (IRB), and based on specialty practice of neuroendocrine oncology at HLMCC, a detailed clinicopathologic analysis and extended follow up study was designed and conducted to assess the clinicopathological charcateristics of primary GEP-PDNECAs patients, and to assess their survival based on conventional chemotherapy regimens. These patients had undergone biopsy / resection at H.Lee Moffit Cancer Center (HLMCC) and Research Institute Tampa; Florida USA or at an outside referring institution. The data sources were pathology archives, electronic patient medical records, neuroendocrine oncologists’ consultation files, institutional tumor registry and social security index. All available slides were reviewed and tumors were histologically sub-typed by expert neuroendocrine pathologists (AN, DC). Subsequently, clinicopathologic data and patient survival were analyzed.

### Inclusion criteria

Adult male and female patients with GEP-PDNECAs, who were treated with systemic platinum and etoposide therapy at HLMCC between 1999 and 2009.

### Exclusion criteria

Patients with incomplete clinical/follow-up data or inadequate non-contributory immunohistochemical marker studies were excluded.

We correlated tumor site, patient age, sex, histological typing and treatment with overall survival. In sub-specialty neuro-endorine oncologic pathology setting, we also reviewed all available biopsy/resection specimen slides of all 68 patients, including hematoxylin and eosin stains, tumor mitotic rate and immunostains for synapnophysin, chromogranin and CD56 and rendered final diagnosis and interpretation of all surgical pathology specimens.One, three, five and ten year survival was analyzed with reference to clinicopathologic parameters in all patients.

In small cell variant of poorly differentiated neuroendocrine carcinoma, morphologically their cells were small with scant cytoplasm, fine chromatin, nuclear molding, and diffuse pattern of growth. There were numerous mitotic figures (by definition >10/10HPF), with Ki-67 index ≥25%, abundant necrosis. Immunohistochemically, they were positive for synaptophysin and/or chromogranin/CD56, either focally of diffuse. In large cell variant of poorly differentiated neuroendocrine carcinoma, there were more prominent nesting pattern, cells with moderate amount of amphophilic cytoplasm, large nuclei with clumped chromatin and prominent nucleoli. Numerous mitotic figures (by definition >10/10HPF) and positive staining with neuroendocrine markers was required for their diagnosis and Ki-67 index was ≥25%.^12^,^13^

Data was analyzed by using SPSS 17. The significant level was calculated using conventional criteria considering the level significant when less than 0.005. Confidence intervals and intermediate values were used in calculations, after calculating means, standard deviations, standard errors, and difference of grades between the two groups. Pearson correlation test, and Spearman correlation test were used to evaluate the association between variables when appropriate.^14^

## RESULTS

This study included 41 males and 27 females with the age range 25-76 yrs. Their mean age was 42 yrs. The majority of the tumors were from colorectal region 39/68 (57.35%) and pancreas 19/68 (28%). While 4/68 (6%) cases were from small intestine (SI), 3/68 (4%) cases from stomach. Only 3/68 (4%) cases were having multiple origin i.e., colon/SI/pancreas (Table-I-III).

**Table-I:** Histological spectrum of gastroenteropancreatic –poorly differentiated neuroendocrine carcinoma.

Lesions	SCC	LCC	`Mixed (NEC) With NED	PD	Total
Stomach	2	1	0	0	3
Small Intestine	2	2	0	0	4
Colon	16	10	2	4	32
Rectum	4	2	1	0	7
Pancreas	13	1	2	3	19
Mixed (Colon+ Pancreas + SI)				3	3

Total	37 (54.5%)	16 (23.5%)	5 (7.3%)	10 (14.7%)	68

***Note:*** Key: PD+NE: Poorly differentiated carcinoma with neuroendocrine carcinoma, SI: small intestine, SCC: small cell carcinoma, LCC: large cell carcinoma, Mixed NEC: mixed small cell and large cell carcinoma, NED with neuroendocrine differentiation.

**Table-II:** Positivity of Immunohistochemistry for different Markers in Gastroenteropancreatic Poorly Differentiated Neuroendocrine Carcinoma.

Tumors	Chromo (n=65)	Synapt (n=67)	CD56 (n=21)	One of Markers	Two of Markers	Three of Markers
SCC	24	29	9	11	19	3
LCC	9	19	4	9	9	3
Mixed	1	5	2	1	4	2
PDC with NEF	4	9	2	1	8	1

Total	38 (54.5%)	62 (92.5%)	17 (81%)	22 (32%)	40 (59%)	9 (13%)

Key’ Chromo: chromogranin, Synap: synaptophysin.

***Note:*** none of the case was negative for any one of the above-mentioned immunomarker.

**Table-III:** A ten year Survival of Patients of Gastroenteropancreatic Poorly Differentiated Neuroendocrine Carcinoma; following Carboplatin (Platinum) and Etoposide therapy.

Years	Sex		Total	Percentage

	Male	Female		
<1	41	27	68	100
1	33	25	58	85
2	18	16	34	50
3	14	13	27	40
4	9	7	16	23.5
5	7	6	11	16
6	4	3	7	10
7	2	2	4	6
8	0	2	2	3
9	0	2	2	3
10	0	1	1	1.5
>10	0	1		1.5

There were 63/68 (93%) patients who presented with lymph node/distant metastases. Regarding the histological categorization, of 68 tumors, 37 (54%) were classified as small cell carcinoma (SCCA), 16 (24%) large cell carcinoma (LCCA), 5 (7%) mixed small and large cell (MSLCCA) and 10 (15%) poorly differentiated carcinoma with neuroendocrine features (PDCA-NEF) (Table-I).

Tumors were positive for chromogranin in 38/65 (55%), synaptophysin in 62/67 (92%), and CD56 in 17/21 (81%) cases. One marker was positive in 22/68 (32%), 2 in 40/68 (59%) and all 3 were positive in 9/68 (13%) cases (Table-II).

**Fig.1:**
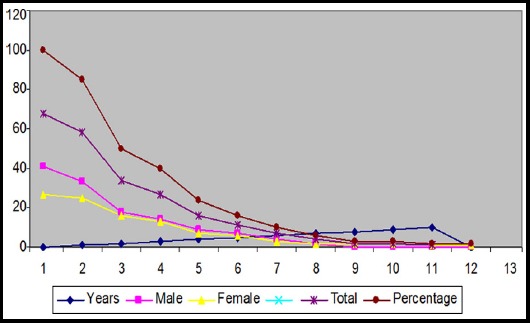
Survival of male and female patients with Gastroenteropancreatic Poorly Differentiated Neuroendocrine Carcinoma, following Carboplatin & Etoposide therapy.

Fifty-eight of 68 (85%) patients were treated with platinum and etoposide. Overall survival at 1, 3, 5, and 10 years was 85%, 40%, 16% and 1.5% respectively. Patient survival was independent of age (r= 0.1022), sex (r= -0.909) and histologic subtype (r=0.1028) (p= 0.128) but was related to distant metastases (r=0.306; p=0.0383) (Table-III and Fig.1).

r= -0.909, (Sex) r= -0.1022 (age), r= -0.1028 (histological types); P=>0.5 for each variable

## DISCUSSION

Neuroendocrine gastroenteropancreatic tumors are not common and constitute 2% to 3% of all gastrointestinal malignancies while poorly differentiated neuroendocrine carcinomas constitute less than 1% of all NETs of the gastroenteropancreatic system.^15^,^16^ There is a rising incidence in most subpopulations possibly related to improved compliance with surveillance colonoscopies and improved endoscopic and radiographic techniques. Further studies are needed to ultimately determine the exact cause of such findings.^17^

Gastroenteropancreatic Neuroendocrine Neoplasms (GEP-NENs) are two genetically different entities, well-differentiated neuroendocrine tumours (NETs) and poorly differentiated neuroendocrine carcinomas (NEC). The poorly differentiated NET or NECs are characterized by a dismal prognosis due to mutation and inactivation of tumor suppressor genes, TP53, RB1, CDKN1 and APC in NEC of GIT origin and inactivation of MEN1, VHL, TSC1/2, and the hyperactivation of the PI3K/mTOR pathway as distinctive biological features of these neoplasms of NEC arising from Pancreas.^18^,^19^

The ileum, appendix, and rectum are the most common sites of involvement followed by colon, stomach, and duodenum. The most important criteria of malignancy in pancreatic NETs are tumor size (>2 cm), angioinvasion, proliferative activity (>2%), invasion of adjacent organs, and metastases to the regional lymph nodes and liver.^20^

The presenting symptoms are nonspecific and consist of abdominal pain, nausea, diarrhea, weight loss, and carcinoid syndrome (10%). The most common site of metastases is regional lymph nodes. Distant metastatic sites include liver (44%), lung (14%), peritoneum (14%), and bone (7%). The Ki-67 score seems to be a better predictor of survival than the degree of differentiation.^20^-^22^

In our study the age range of the patients was 25-76 years with mean age 45 years. This is consistent with a recent study by Ulla Rocha et al (2017).^23^ It is obvious that these tumors occur in younger as well as older age group. This age range is consistent with another study published from Moffitt Cancer Center in 2009 by Strosberg et al. The median age in these patients at diagnosis was 57 years (range 23-83 years).^13^ No statistical sex difference was seen in the patients between present and other studies.

Ours is one of the larger studies on 68 cases of poorly differentiated neuroendocrine carcinoma of gatrointestinopancreatic region, including 39 cases from colorectal region, 19 cases from pancreas, four cases from small intestine, three cases from stomach and three multi-focal cases involving colon, small intestine and pancreas.

Majority of our patients with PD-NECA presented with distance metastases, which was found to be most important prognostic factor in survival of our patients. In our study 93% patients were bearing distant metastasis at the time of diagnosis. These findings are consistent with already reported data.^13^,^24^,^25^

The histological diagnosis was made on the available specimens by examining hematoxylin and eosin stained slides for mitotic rate and immunostaining for measurement of the Ki-67 index. Majority of the cases were diagnosed into three categories according to WHO classification. Three histological types were small cell carcinoma (54%), large cell carcinoma (24%) and carcinoma with mixed features (7%) of SCC and LCC. However, few of the tumors were showing poorly differentiated morphology with neuroendocrine features (15%).

In this study the synaptophysin and CD 56 were found the most supportive immunomarkers for the diagnosis of poorly differentiated neuroendocrine carcinomas as compared to chromogranin. Other markers like, NSE, S100 proteins and Cytokeratins, did not show any specificity in this group of malignancies. Tumors were positive for synaptophysin in 92%, CD56 in 81% and chromogranin in 55%, cases. One marker was positive in 32%, two in 59% and all three were positive in 13% cases. These findings are consistent with similar analyses by Shia J et al (2008) and Mia-Jan et al (2013).^12^,^26^

Fifty-eight of 68 (85%) patients were treated with platinum and etoposide. Overall survival at 1, 3 and 5 years was 85%, 40% and 24% respectively. Regarding the patients’ survival suffering from these poorly differentiated neuroendocrine carcinoma, the outlook was hopeful due to advanced methods of diagnosis and treatment. All the patients survived after 6 months while 85% were alive after 1 year of their diagnosis. Two of our patients survived up to 10 years after first presentation of their disease. One of our patients was still alive beyond 10 years after diagnosis. The survival of our patients was independent of age (r= 0.1022), sex (r=-0.909) and histologic subtype (r=0.1028) (p=0.128) but was related to distant metastases (r=0.306; p=0.0383).

## CONCLUSIONS

GEP-PDNECA cases were diagnosed in any part of the GI tract, most commonly in the colorectal region. Although positivity of neuroendocrine markers in tumor tissues was variable, it was an important adjunct to confirm neuroendocrine differentiation in these high-grade malignancies and to avoid under-diagnosis of GEP-PDNECA, especially in metastatic setting. Overall prognosis of GEP-PDNECA patients following platinum and etoposide therapy in our series was relatively favorable but remained poor in the presence of distant metastases. Mere histopathological examination is not enough for the diagnosis of GEP-PDNECA. A panel of immunohistochemical neuroendocrine markers is required to avoid misdiagnosis of these malignancies. Synaptophysin was the most sensitive marker; however, panels of 2 or 3 neuroendocrine markers (Syn, Cg and CD56) are required for proper diagnosis and Ki-67 index for accurate determination of grade.

## PERSPECTIVE

Although overall survival of GEP-PDNECA patients following platinum and etoposide in our series was relatively favorable with some long-term survivors, with the recent advancement of more personalized therapeutic options, there is need to utilize more effective novel therapies to further improve and sustained survival of patients with these aggressive malignancies.In this regard, novel molecularbiomarkers may play an important role for early diagnosis, localization of recurrence/metastasis and improved clinical response of GEP-PDNECA patients to newer targeted and immunotherapies.

### Authors’ Contribution:

**MHB:** Data collection, pathology review/interpretation, designed and carried statistical analysis, wrote first draft, approved final manuscript, is responsible for integrity of research.

**DC:** Sub-specialty neuroendocrine pathology review/interpretation, data analysis, review, editing and approval of final manuscript.

**AN:** Conceived and designed study, sub-specialty neuroendocrine pathology review/interpretation, data analysis/interpretation, review, editing and approval of final manuscript.
